# A Plea for Education

**Published:** 1884-09

**Authors:** Geo. L. Paine

**Affiliations:** Xenia, O.


### A Plea for Education.

BY GEO. L. PAINE, D.D.S., XENIA, O.

Read before the Mad River Valley Dental Society.

LORD BACON says: "I hold every man a debtor to his profession from the which, as men, of course, do seek to receive countenance and profit, so ought they of duty to endeavor themselves, by way of amends, to be a help and ornament thereunto."

A profession is that of which one professes knowledge—if not mechanical— and to which one devotes himself; the business which one professes to understand, and follows for a subsistence.

The business of a lawyer, or physician, is professional. The learned professions are theology, law, medicine, and dentistry.

Every man, according to our text from Bacon, owes a duty to the profession of which he is a member, *i. e.*, to be to it both helpful and ornamental. To be helpful is to afford assistence, when called upon, and implies that both mental and physical resources are at the command of the helper, whose efficiency can be measured only by the limitations of his education, which is the training of his physical and intellectual powers.

That the best results may be attained in all respects, the physical powers must be trained, thus securing the highest degree of health and consequent physical vigor and efficiency. Without health the most elaborate mental culture is, to a great extent, a failure; thus verifying the truth of the adage: "*Sana mens in corpore sano.*"

Candidates for distinction in all professions, and especially in the dental, should possess, *de natura*, a fine physique, not only to enable them successfully to perform the tasks demanded of them as students in college or university, but also to enable them to endure the arduous labors and nervous tension incident to the practice of their profession.

It is evident, therefore, that a sound and healthy physique is an indispensable factor to him who hopes to win honor and emolument in the practice of a profession.

Having defined what constitutes our ideal candidate for professional life, we proceed to summarize what should constitute his mental outfit, or educational equipment. That every aspirant for professional honors should be well educated before he enters upon the study of his profession proper, is a proposition not likely to be disputed by any one whose opinion is entitled to respect. The demand made by the best men of our times is that those who intend to study any profession be thoroughly drilled in the classics, and, if possible, in German and French, as well as in those branches of science which have a direct bearing on the profession that the student expects to enter.

This education is as essential to the professional man as a knowledge of the manual of arms is to the soldier, as it enables him readily to comprehend the principles, and successfully to master the details of his life work.

The mental as well as the physical faculties demand severe training, and will not do effective work without it. Every faculty of the mind may be likened to a tool which one must be taught how to use, and to keep in good order.

One may be said to be educated when his faculties, like well drilled soldiers, respond promptly to the calls of duty, and are always ready for active and useful service.

Our candidate, with a training such as we have outlined, is ready to enter upon the study of his chosen profession. Other things being equal, he will gain a more comprehensive knowledge of his profession, than a comparatively uneducated person, and gain it more easily.

Thus, with educated head and hand, he enters upon his life-work; and how great the fruitage!

How transcendently beautiful are the sculptured creations of a Powers or a Canova, or the paintings of a Rembrandt or a Titian, with cultivated brains guiding the chisels of the one and the brushes of the other!

So, by parity of reasoning, we may infer that, when natural ability is supplemented by the skill imparted by education, and our efforts are directed by studious and scholarly men, the results must be correspondent, not only to the text of my Lord Bacon, but also to the ideals of the most artistic and poetic of our profession.

